# Diagnostic Test Precision of Modified Carbapenem Inactivation Method and Carbapenemase Nordmann-Poirel Test for Phenotypic Detection of Carbapenemase Production in Enterobacterales: A Systematic Review

**DOI:** 10.7759/cureus.67322

**Published:** 2024-08-20

**Authors:** Vijay Suriya R, Leela KV, Han Feliciana J, Aishwarya R

**Affiliations:** 1 Microbiology, SRM (Sri Ramaswamy Memorial) Medical College Hospital and Research Centre, SRM Institute of Science and Technology, Kattankulathur, IND

**Keywords:** modified carbapenem inactivation method, meropenem disk inactivation, colorimetric assay, ambler classification, clsi, carbapenemase producing enterobacterales, carbapenemase, carbapenem resistant enterobacterales, mcim, carba np

## Abstract

Carbapenem-resistant Enterobacterales, particularly those that produce carbapenemases, pose a significant public health concern due to very limited treatment options. The timely identification of carbapenemase-producing Enterobacterales (CPE) is essential for putting in place efficient infection control measures and selecting appropriate antimicrobial therapies, thereby improving the clinical outcome of the patient. The purpose of this systematic review is to compare the diagnostic accuracy and practicality between two phenotypic tests, namely the modified carbapenem inactivation method (mCIM) and carbapenemase Nordmann-Poirel (Carba NP) test, in detecting carbapenemase production by Enterobacterales and thereby aiding the clinician in making a decision to choose an appropriate test for their phenotypic detection.

This systematic review involved combining sensitivity, specificity, positive predictive value (PPV), negative predictive value (NPV), accuracy, diagnostic odds ratio with 95% confidence interval (CIs), Forest plot for sensitivity and specificity, and plotting suitable summary receiver operating characteristic curve with the area under the curve.

Of the 20 studies included in this review, the overall effect sizes of Carba NP and mCIM with 95% CIs were as follows: sensitivity, 91% (86-96%) and 97% (95-99%); specificity, 93% (88-97%) and 97% (93-100%); PPV, 97% and 98%; NPV, 79% and 90%; accuracy, 93% and 97%; diagnostic odds ratio, 1487.8879 and 8527.5541; and AUC, 0.85 and 1, respectively.

In conclusion, the mCIM method showed superior sensitivity (97%), specificity (97%), and accuracy compared to the Carba NP test in detecting carbapenemase production, even though both these methods had a few technical limitations. The Carba NP test is rapid, affordable, and dependable, whereas mCIM is more accurate and cost-effective but time-consuming. We propose that both tests can be reliably used for screening of carbapenemase production in Enterobacterales, as endorsed by the Clinical and Laboratory Standards Institute even in resource-limited clinical laboratories, in the order of prioritizing the mCIM method first and then followed by the Carba NP test when situation demands expedited results.

## Introduction and background

In healthcare settings, carbapenem-resistant Enterobacterales (CRE) are significant pathogens causing severe infections with high morbidity and mortality rates [1]. Various studies have indicated that carbapenem-resistant organisms (CROs) are linked to mortality rates that are four times higher compared to that of carbapenem-susceptible organisms [2]. The global spread of CRE has been extensively documented over recent years [3,4]. With the recent advancements in laboratory technologies, identifying the resistance mechanisms in CRE infections is crucial for administering precise antimicrobial treatment.

Resistance to carbapenems encompasses various mechanisms such as upregulation of efflux systems, loss of the outer membrane porins, and most notably enzymatic degradation by the production of carbapenemases [5]. Carbapenemases are categorized by Ambler classification under the following classes: class A (KPC, SME, GES), class B (VIM, NDM, IMP), and class D (bla_OXA-48_, bla_OXA-48_ like) [6]. These CRE can be further categorized based on their ability to produce carbapenemase into carbapenemase-producing Enterobacterales (CPE) and non-carbapenemase-producing Enterobacterales (non-CPE). Among these, the carbapenemase producers are particularly concerning due to their higher virulence and increased potential for horizontal transmission, posing greater risks to the patients and healthcare system [7].

Since the emergence of CPE, researches on the methods for phenotypic detection have expanded steadily [8]. These include the combined disk tests , modified Hodge test, carbapenemase Nordmann-Poirel (Carba NP) test and its variants, modified carbapenem inactivation method (mCIM), agar-based culture media with antibiotics, and automated identification systems [9]. Nevertheless, there is no single gold standard phenotypic detection method that fulfills all the requirements flawlessly. This systematic review aims to analyze two phenotypic detection methods, namely mCIM and Carba NP test, which are the only phenotypic detection methods currently endorsed by the Clinical and Laboratory Standards Institute (CLSI) for detection of carbapenemase production [10,11]. The objectives of this review are to assess the accuracy of these phenotypic detection methods and to evaluate their practicality considering economic factors and clinical efficiencies.

## Review

Methodology

Literature Search Process

An extensive literature search was performed on multiple databases, such as PubMed and Google Scholar, up until July 2024. The keywords for the search included "carbapenemase-producing Enterobacterales" OR "carbapenemase-producing Enterobacteriaceae" AND "Carba NP" AND "mCIM". The search was limited by the date of publication between January 2018 and July 2024 and not filtered in terms of testing method, country, or research design. Additionally, references from the retrieved studies were manually reviewed.

Selection Criteria

Criteria for inclusion: The included studies evaluated the diagnostic precision of two phenotypic tests, namely mCIM and Carba NP, for the detection of CPE. The exclusion criteria for this study were as follows: Studies that were published before the year 2018 were excluded and articles published in languages other than English and those studies in which the full text could not be retrieved were excluded. Studies in which sensitivity and specificity outcomes were not analyzed. Organisms other than Enterobacterales, such as carbapenemase-producing *Pseudomonas aeruginosa* or *Acinetobacter* spp., were excluded. Additionally, studies that only analyzed one type of carbapenemase genotype and those that assessed only one phenotypic method either mCIM or Carba NP and not both were also excluded

Extraction Toolkit

A data extraction toolkit was developed to gather information on several aspects, including the first author, publication year, study design, sample size, and the methodologies employed for detection along with their corresponding sensitivity and specificity. To maintain uniformity in the screening criteria and data collection, three authors conducted a thorough examination of the literature and retrieved the necessary data. The risk of bias in the selected studies was very low as we included articles with only high scores as provided by the standard quality assessment tools. Any differences that arose were settled through thorough deliberation and by engaging a mediator until a consensus was reached.

Statistical Analysis and Data Integration

Using SPSS v.29 (Armonk, NY: IBM Corp), the following metrics were assessed: sensitivity, specificity, positive predictive value (PPV), negative predictive value (NPV), diagnostic odds ratio, and accuracy. Summary receiver operating characteristic (SROC) curve associated with 95% CI for the mCIM and Carba NP test in detecting carbapenemase production in Enterobacterales was plotted along with AUC. A p-value below 0.05 was deemed to have statistical significance.

Results

Summary of Eligible and Incorporated Research

**Figure 1:**
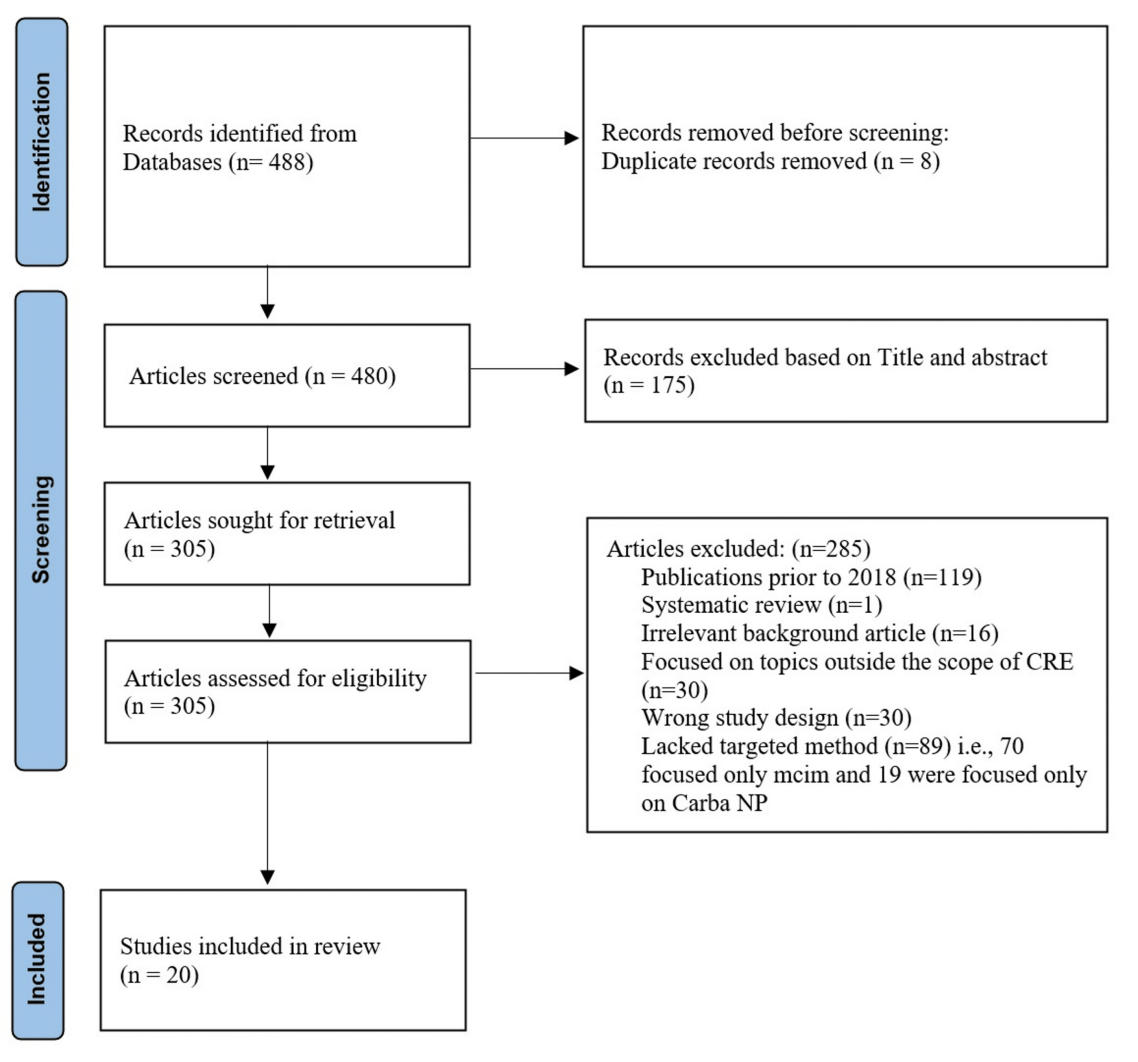
PRISMA chart for retrieval of included articles CRE: carbapenem-resistant Enterobacterales; Carba NP: carbapenemase Nordmann-Poirel; mCIM: modified carbapenem inactivation method.

The search method yielded 488 potentially relevant publications. After removing articles based on their titles, abstracts, and eight duplicated articles, 305 articles were subjected to an initial full-text screening process. Among them, 285 research were subsequently excluded, since 119 of them were published prior to the year 2018; one was a systematic review; 16 had an irrelevant background article; 30 focused on topics outside the scope of CRE; 30 had wrong study design; 89 lacked targeted methods (i.e., 70 focused only mCIM and 19 were focused only on Carba NP). Ultimately, 20 articles were included in the analysis, consisting of studies comparing both mCIM and Carba NP [12-31]. The literature inclusion process is depicted in Figure 1.

Assessment of Diagnostic Efficacy of the Tests

**Figure 2:**
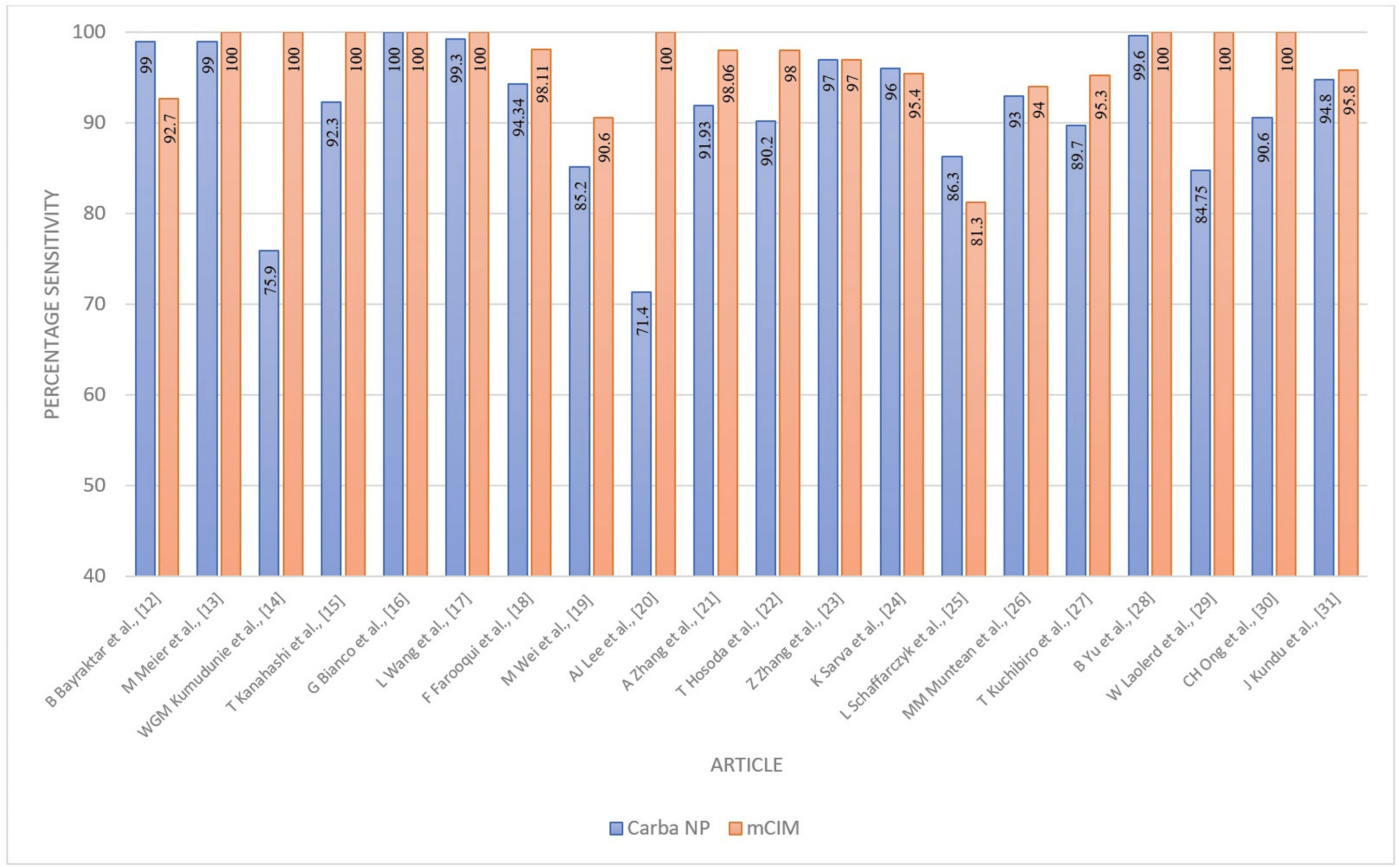
Performance characteristics - sensitivity of Carba NP versus mCIM Carba NP: carbapenemase Nordmann-Poirel; mCIM: modified carbapenem inactivation method.

**Figure 3:**
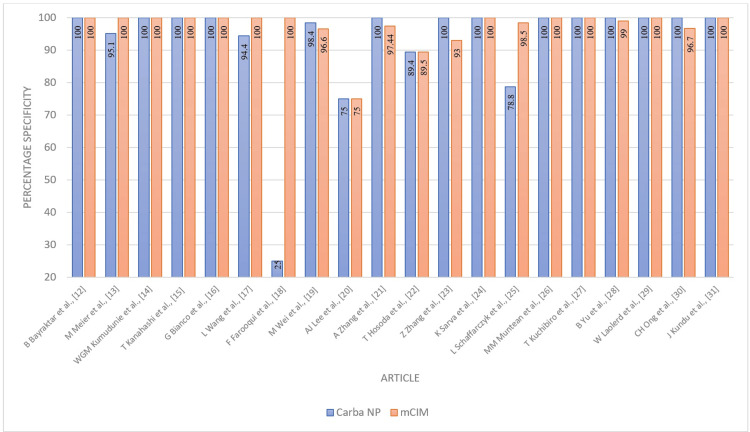
Performance characteristics - specificity of Carba NP versus mCIM Carba NP: carbapenemase Nordmann-Poirel; mCIM: modified carbapenem inactivation method.

**Figure 4:**
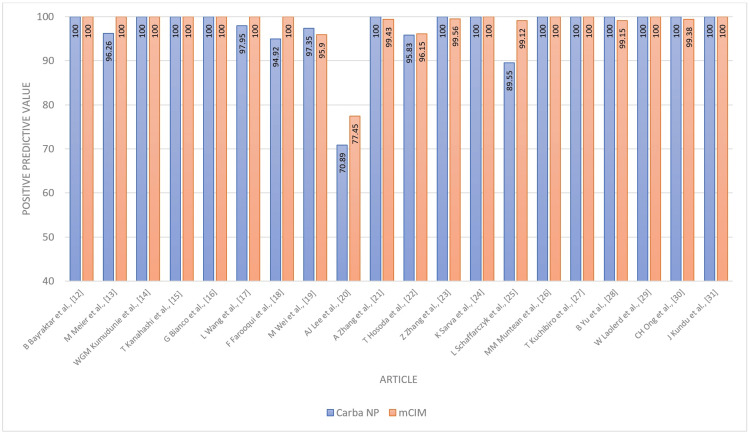
Positive predictive value - Carba NP versus mCIM Carba NP: carbapenemase Nordmann-Poirel; mCIM: modified carbapenem inactivation method.

**Figure 5:**
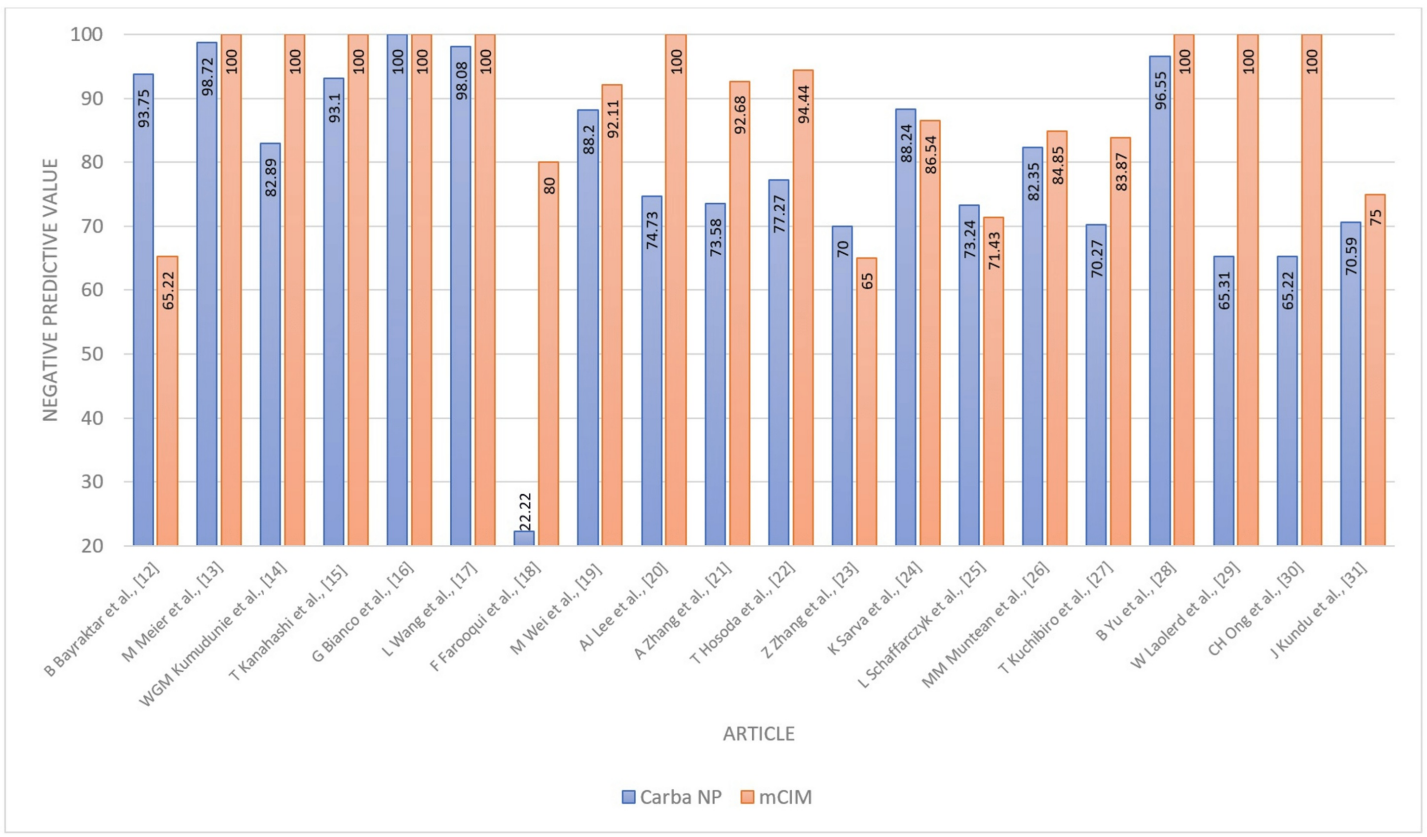
Negative predictive value - Carba NP versus mCIM Carba NP: carbapenemase Nordmann-Poirel; mCIM: modified carbapenem inactivation method.

**Figure 6:**
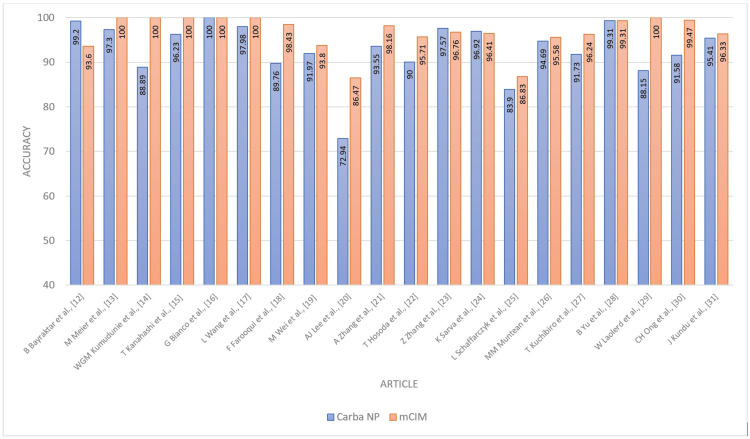
Accuracy - Carba NP versus mCIM Carba NP: carbapenemase Nordmann-Poirel; mCIM: modified carbapenem inactivation method.

**Figure 7:**
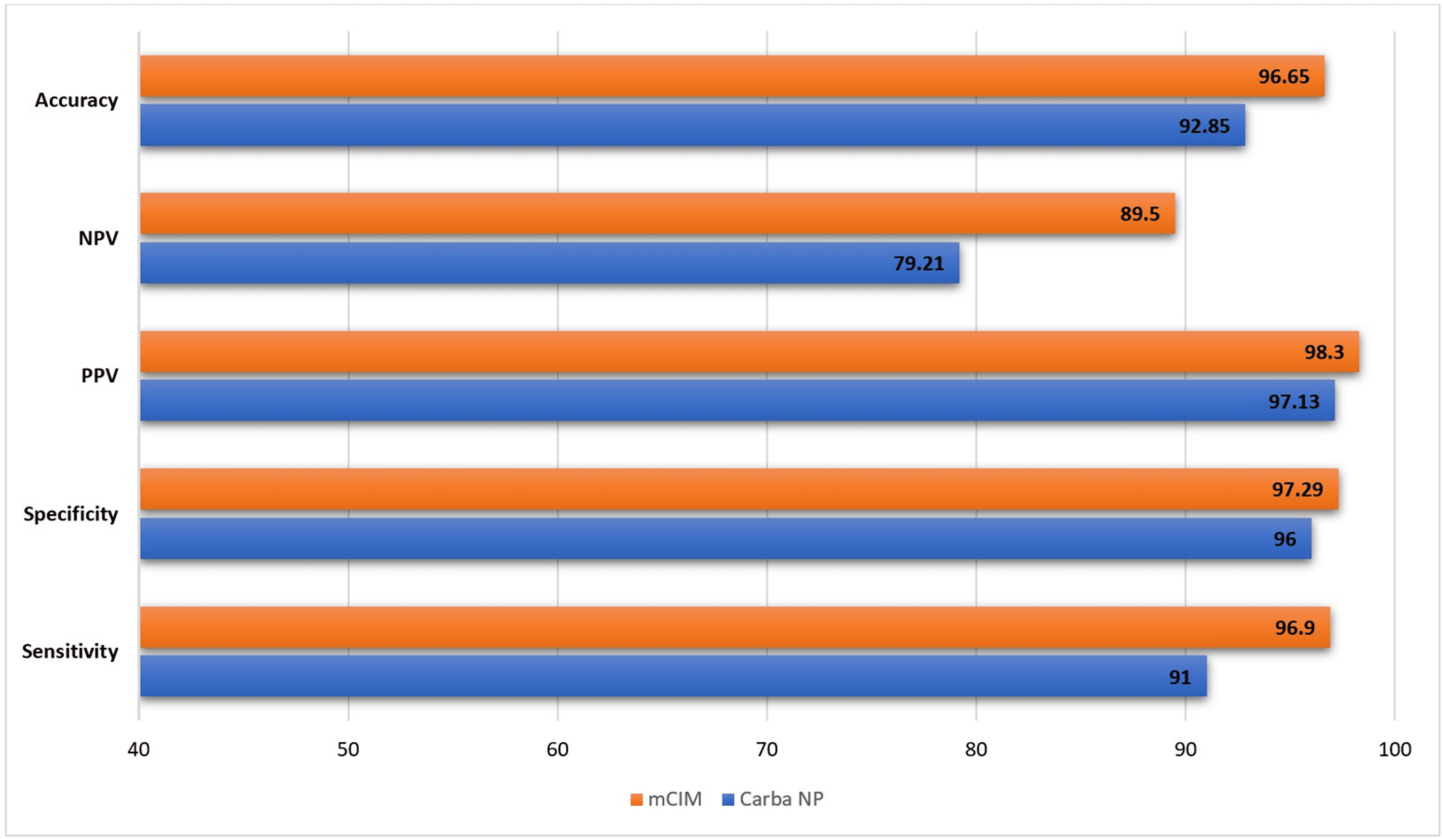
Integrated analysis - Carba NP versus mCIM Carba NP: carbapenemase Nordmann-Poirel; mCIM: modified carbapenem inactivation method; PPV: positive predictive value; NPV: negative predictive value.

The Carba NP test demonstrated an overall sensitivity of 91% (95% CI 86-96%) and specificity of 93% (95% CI 88-97%). These performance characteristics are plotted in Figures 2, 3. The combined averages of PPV, NPV, accuracy, and odds ratio were 97%, 79%, 93%, and 1487.8879, respectively, with AUC 0.85 (Figures 4-7).

The mCIM technique demonstrated an overall sensitivity of 97% (95% CI 95-99%) and specificity of 97% (95% CI 93-100%) (Figures 2, 3).

The combined averages of PPV, NPV, accuracy, and odds ratio were 98%, 90%, 97%, and 8527.5541, respectively, with an AUC of 1 (Figures 4-7).

**Figure 8:**
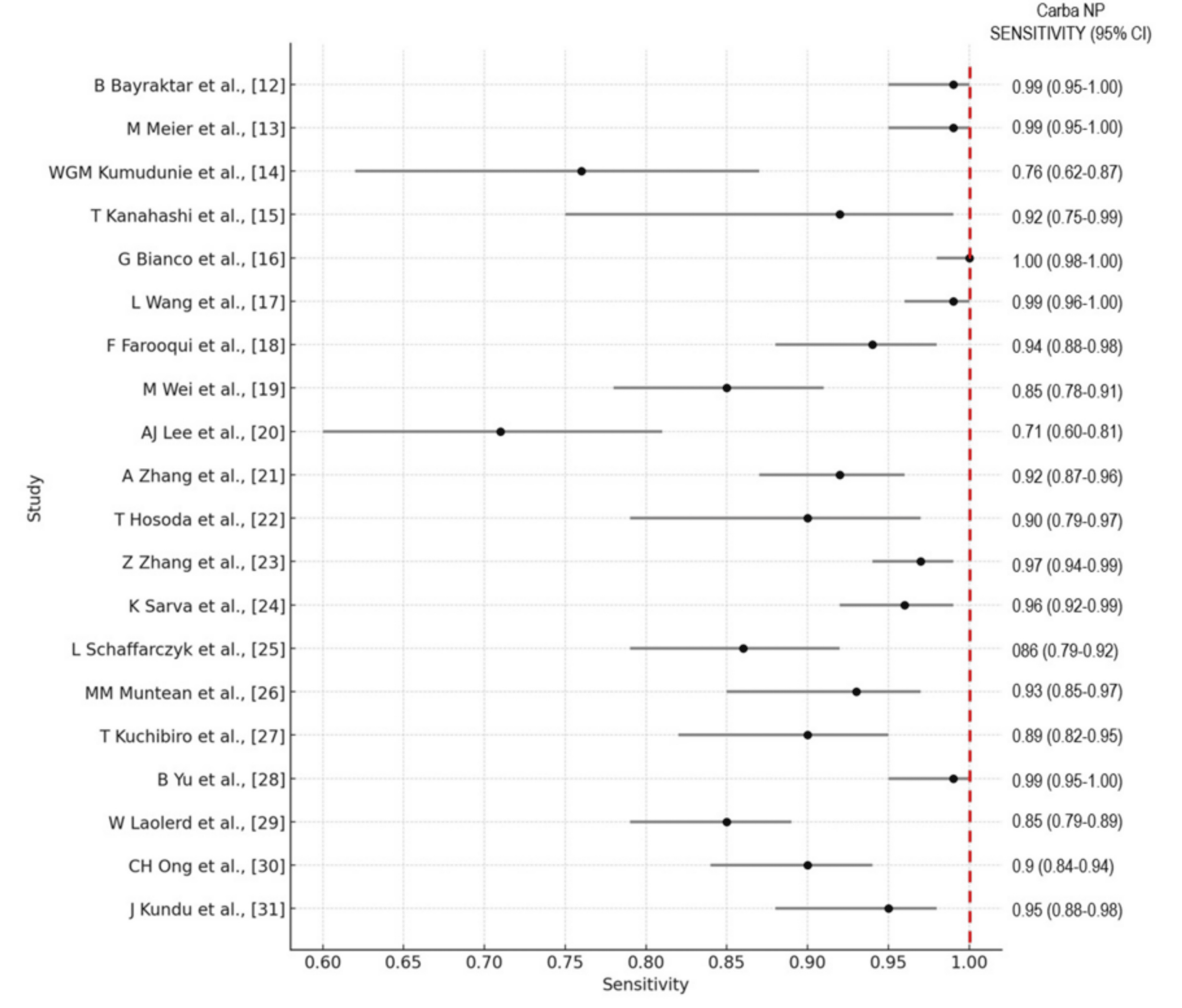
Forest plot illustrating the overall performance of the Carba NP in terms of sensitivity with 95% CIs. Carba NP: carbapenemase Nordmann-Poirel; CI: confidence interval.

**Figure 9:**
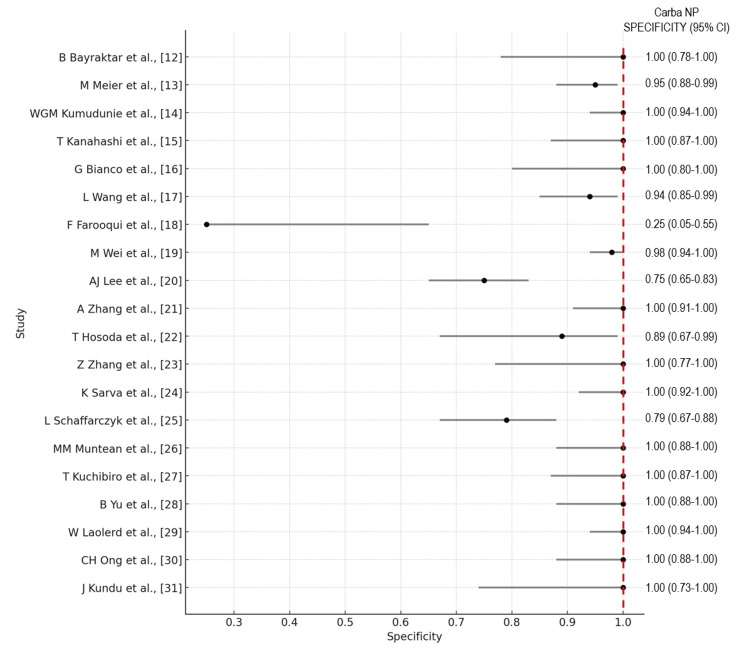
Forest plot illustrating the overall performance of the Carba NP in terms of specificity with 95% CIs. Carba NP: carbapenemase Nordmann-Poirel; CI: confidence interval.

**Figure 10:**
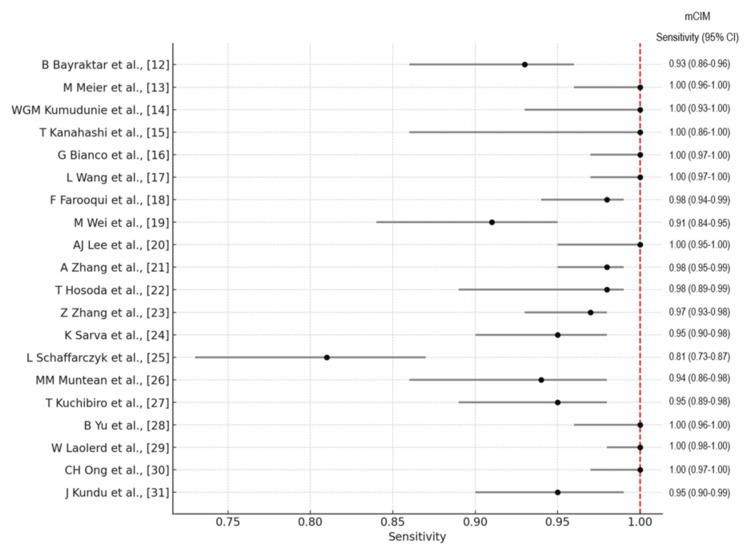
Forest plot illustrating the overall performance of the mCIM in terms of sensitivity with 95% CIs. mCIM: modified carbapenem inactivation method; CI: confidence interval.

**Figure 11:**
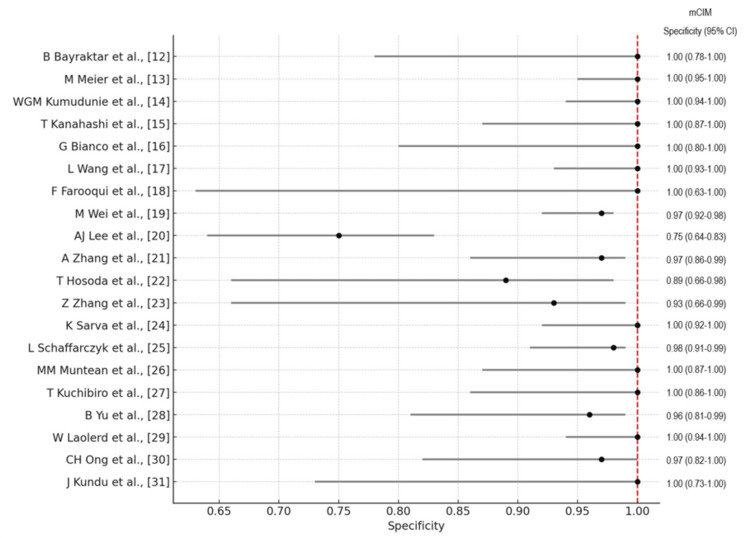
Forest plot illustrating the overall performance of the mCIM in terms of specificity with 95% CIs. mCIM: modified carbapenem inactivation method; CI: confidence interval.

The forest plots of the overall performance of Carba NP and mCIM in terms of sensitivity and specificity with 95% CIs are illustrated in Figures 8-11, respectively. 

**Figure 12:**
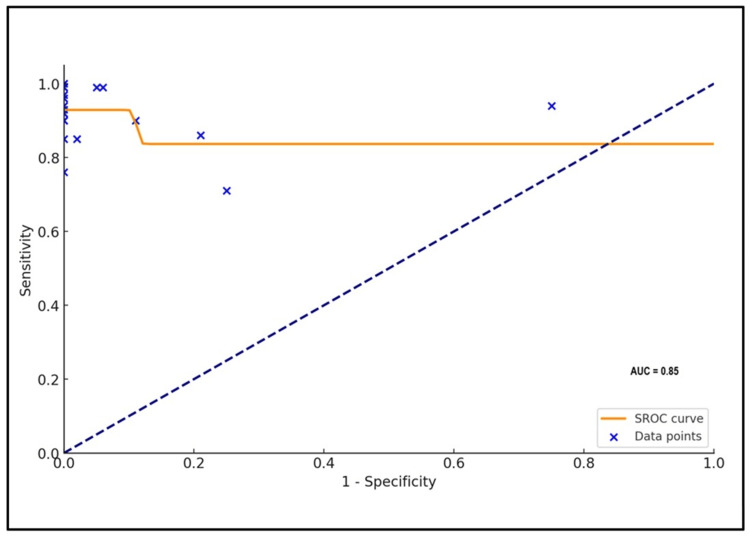
Summary receiver operating characteristic curve of Carba NP. AUC: area under the curve; SROC: summary receiver operating characteristic; Carba NP: carbapenemase Nordmann-Poirel.

**Figure 13:**
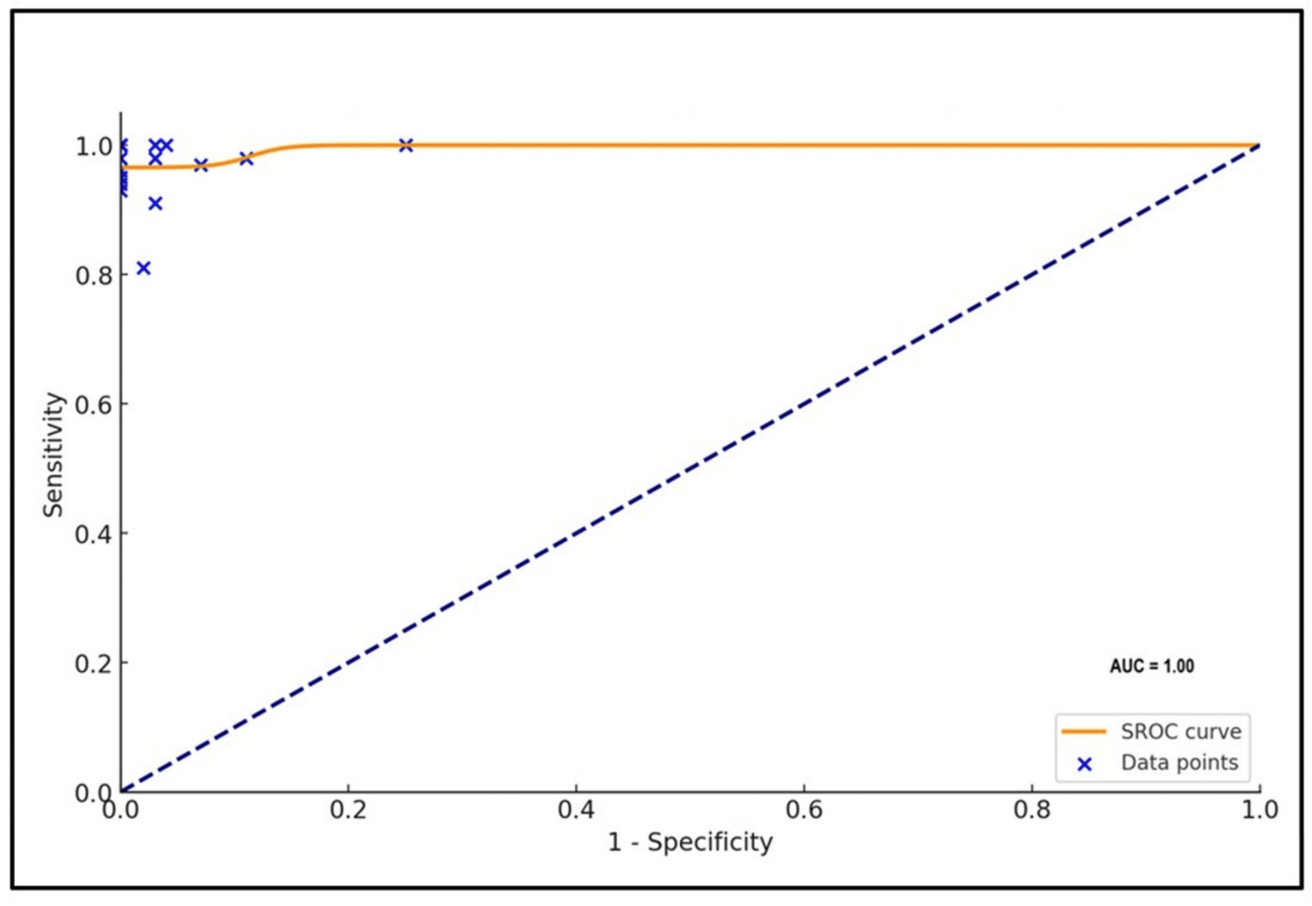
Summary receiver operating characteristic curve of mCIM. AUC: area under the curve; SROC: summary receiver operating characteristic; mCIM: modified carbapenem inactivation method.

The SROC curve analyses with the area under curve for Carba NP and mCIM methods are plotted in Figures 12, 13, respectively. 

Comparison of Phenotypic Methods Based on Their Characteristics

The characteristics of Carba NP and mCIM are summarized based on their principle, requirements, turnaround time, result interpretation, accuracy, and their testing expenditure in Table 1.

**Table 1:** Summary of characteristic features of Carba NP and mCIM. Carba NP: carbapenemase Nordmann-Poirel; mCIM: modified carbapenem inactivation method; ZnSO_4_.7H_2_O: zinc sulfate heptahydrate; NaOH: sodium hydroxide; INR: Indian rupee.

Characteristic features	Carba NP	mCIM
Principle	Colorimetric microtube assay	Meropenem disk inactivation
Requirements	ZnS0_4._7H_2_O, phenol red, NaOH, solution A and solution B(with imipenem)	Tryptone soya broth, meropenem disk
Turnaround time	2 hours	18-24 hours
Result interpretation	Observing color change	Measuring the zone of inhibition
Sensitivity and specificity	91%; 93%	97%; 97%
Cost (INR)	≈144	<72
Limitations	Reagents are sensitive to light and should be stored at 4-8°c and have short shelf life. In certain instances, false-negative outcomes were observed when the colonies exhibited mucoid characteristics. Some tests yield invalid results.	Only applies to Enterobacterales and *Pseudomonas aeruginosa*. Time consuming and not quantitative.

Discussion

Carba NP, a rapid colorimetric microtube assay which is based on the hydrolysis of carbapenem by the carbapenemase-producing organisms, demonstrated an overall sensitivity of 91% and specificity of 93% with its potential to differentiate between carbapenemase-producing bacteria and carbapenem-resistant organisms that exhibit other resistance mechanisms [32]. The pooled specificity could have been augmented to approximately 96% if not for one study [18] where the specificity was as low as 25% bringing down the overall average of specificity to 92%. Moreover, Carba NP exhibited a low sensitivity for blaOXA-48 and blaOXA-48-like β-lactamases because of their low enzymatic activity [29,30]. Additionally, few studies showed low sensitivity for the Carba NP test with those organisms producing mucoid colonies and Enterobacterales harboring GES (Guiana extended-spectrum beta-lactamase) carbapenemase [33]. For Carba NP, the "method" covariate effect was observed, with better outcomes when adhering to the CLSI guidelines compared to using a paper strip or an agar-based medium.

The mCIM is based on the principle of carbapenem disk inactivation, where the carbapenemase-producing bacteria degrades the carbapenem disk and thereby reduces the zone of inhibition when placed on to a carbapenem-sensitive *Escherichia coli* ATCC 25922 strain lawn culture. Despite the longer incubation duration of 4 hours (alongside overnight incubation for final interpretation) and the use of tryptic soy broth instead of saline during the inactivation step [34], the mCIM demonstrated enhanced diagnostic accuracy in detecting CPEs compared to the Carba NP test. In this analysis, the mCIM demonstrated a pooled sensitivity of 96.95% and a pooled specificity of 97.14%.

Both these phenotypic detection methods have their advantages and disadvantages concerning quality control, shelf life, and turnaround time. mCIM is cost-effective and requires no special reagents or equipment, but due to the requirement of overnight incubation the turnaround time is prolonged. Carba NP is relatively more expensive because it necessitates the use of reagents which should be freshly prepared since they have a limited duration of usability.

Early detection of these CRE and distinguishing between carbapenemase producers and non-carbapenemase producers might improve the clinical outcome of the patient and save time and cost amidst the rising prevalence of CPE [8,35]. In the current scenario, phenotypic methods are unanimously recommended for routine screening purposes rather than expensive PCR-based methods that cannot detect novel unidentified genes [9]. On account of a thorough examination of these phenotypic detection methods for CPE, we have determined that the most effective screening method is to prioritize the use of the mCIM method first, followed by the Carba NP test in that order of their efficacy in diagnosis.

Limitations

Nevertheless, a holistic approach should be exercised when interpreting the effectiveness of this systematic analysis, as it has a few limitations. Specifically, the research did not incorporate combined effect sizes for some carbapenemase genes, resulting in equivocal findings regarding the effectiveness of these approaches at the molecular level. The lacunae of unpublished studies and language constraints introduces biases, including language bias and publication bias. Notable heterogeneities observed in some results remain unexplained despite exploring the potential causes. Further studies are needed to comprehensively analyze the diagnostic abilities of these techniques, by enforcing direct comparison or network-based analytical studies inclusive of the patient outcome factor and designing appropriate antimicrobial agents thereby improving the prognosis of the patient.

Notably, a meta-analysis conducted in 2019, which examined papers up until March 2019, stated that the sensitivities of Carba NP and mCIM were 97% (94-98%) and 99% (99-100%), respectively. The specificities of Carba NP and mCIM were 100% (99-100%) and 99% (96-100%), respectively [36].

Compared to the previous systematic review cited above, there is a minimal decline in the overall sensitivity and specificity in this current review for both the phenotypic detection methods Carba NP and mCIM. This difference could have been attributed to various factors such as the following: i) Pathogens can evolve over time, leading to changes in their genetic makeup or expression profiles. ii) The current study population might differ in terms of demographics, immune status, or exposure history compared to the population in which the method was previously validated. iii) Variations in laboratory techniques, reagents, equipment, or protocols over time can affect the performance of diagnostic tests. Even a minor change in procedures or environmental conditions can lead to differences in test outcomes. iv) Due to the appearance of novel variants or strains of pathogens, the method's reliance on phenotypic traits for identification may no longer accurately represent the majority of circulating strains. This discrepancy could potentially reduce the accuracy and precision of the test. v) Lapses in quality control methods, reagents, or equipment performance issues or subjective interpretation in phenotypic assays can influence the test reliability and reproducibility.

## Conclusions

In this systematic review targeted at comparing the precision between two phenotypic methods in detecting carbapenemase production by Enterobacterales, the mCIM method showed a superior sensitivity, specificity, accuracy, and applicability compared to the Carba NP test in a majority of the studies, while some studies showed only a trivial difference in values, even though both these methods had a few technical limitations. As discussed, the Carba NP test is a more rapid, dependable method but necessitates the use of freshly prepared reagents with shorter shelf life, whereas mCIM is more accurate and cost-effective but time-consuming. To conclude, despite very few constraints, we suggest that both tests can be used for screening of carbapenemase production in Enterobacterales but prioritizing the benefits of implementation of the mCIM method over the Carba NP test in resource-limited clinical laboratories. To determine the specific reason for the reduced sensitivity and specificity of the phenotypic detection methods in the current review compared to the earlier studies, a detailed analysis comparing the current conditions with those under which the method was originally validated would be necessary which has been routinely conducted and updated in the CLSI guidelines. This could involve a reassessment of the method's performance characteristics, validation against current standard protocols, and consideration of any changes or developments in the field since its initial validation. Moreover, this review highlights the need for innovation and implementation of newer phenotypic detection methodologies that are better aligned and suitable for contemporary laboratory diagnostics thereby yielding greater benefits.
